# Factors Associated With Racial/Ethnic Group–Based Medical Mistrust and Perspectives on COVID-19 Vaccine Trial Participation and Vaccine Uptake in the US

**DOI:** 10.1001/jamanetworkopen.2021.11629

**Published:** 2021-05-27

**Authors:** Hayley S. Thompson, Mark Manning, Jamie Mitchell, Seongho Kim, Felicity W. K. Harper, Sheena Cresswell, Kristopher Johns, Shoma Pal, Brittany Dowe, Madiha Tariq, Nadia Sayed, Lisa M. Saigh, Lisa Rutledge, Curtis Lipscomb, Jametta Y. Lilly, Heidi Gustine, Annie Sanders, Megan Landry, Bertram Marks

**Affiliations:** 1 Karmanos Cancer Institute, Department of Oncology, Wayne State University School of Medicine, Detroit, Michigan; 2 Karmanos Cancer Institute, Department of Oncology, Wayne State University School of Medicine, Detroit, Michigan; 3 University of Michigan School of Social Work, Detroit; 4 ACCESS, Dearborn, Michigan; 5 St Patrick Senior Center, Detroit, Michigan; 6 Western Wayne Family Health Center, Inkster, Michigan; 7 LGBT Detroit, Detroit, Michigan; 8 Detroit Parent Network, Detroit, Michigan; 9 Area Agency on Aging of Northwest Michigan, Traverse City; 10 United Way of Gratiot & Isabella Counties, Mt Pleasant, Michigan; 11 American Cancer Society–North Central Region, Southfield, Michigan; 12 Faith-Based Genetic Research Institute, Detroit, Michigan

## Abstract

**Importance:**

The impact of COVID-19 in the US has been far-reaching and devastating, especially in Black populations. Vaccination is a critical part of controlling community spread, but vaccine acceptance has varied, with some research reporting that Black individuals in the US are less willing to be vaccinated than other racial/ethnic groups. Medical mistrust informed by experiences of racism may be associated with this lower willingness.

**Objective:**

To examine the association between race/ethnicity and rejection of COVID-19 vaccine trial participation and vaccine uptake and to investigate whether racial/ethnic group–based medical mistrust is a potential mediator of this association.

**Design, Setting, and Participants:**

This cross-sectional survey study was conducted from June to December 2020 using a convenience sample of 1835 adults aged 18 years or older residing in Michigan. Participants were recruited through community-based organizations and hospital-academic networks.

**Main Outcomes and Measures:**

Separate items assessed whether respondents, if asked, would agree to participate in a research study to test a COVID-19 vaccine or to receive a COVID-19 vaccine. Participants also completed the suspicion subscale of the Group-Based Medical Mistrust Scale.

**Results:**

Of the 1835 participants, 1455 (79%) were women, 361 (20%) men, and 19 (1%) other gender. The mean (SD) age was 49.4 (17.9) years, and 394 participants (21%) identified as Black individuals. Overall, 1376 participants (75%) reported low willingness to participate in vaccine trials, and 945 (52%) reported low willingness to be vaccinated. Black participants reported the highest medical mistrust scores (mean [SD], 2.35 [0.96]) compared with other racial/ethnic groups (mean [SD] for the total sample, 1.83 [0.91]). Analysis of path models revealed significantly greater vaccine trial and vaccine uptake rejection among Black participants (vaccine trial*: B* [SE], 0.51 [0.08]; vaccine uptake: *B* [SE], 0.51 [0.08]; both *P* < .001) compared with the overall mean rejection. The association was partially mediated by medical mistrust among Black participants (vaccine trial: *B* [SE], 0.04 [0.01]; *P* = .003; vaccine uptake: *B* [SE], 0.07 [0.02]; *P* < .001) and White participants (vaccine trial: *B* [SE], −0.06 [0.02]; *P* = .001; vaccine uptake: *B* [SE], −0.10 [0.02]; *P* < .001).

**Conclusions and Relevance:**

In this survey study of US adults, racial/ethnic group–based medical mistrust partially mediated the association between individuals identifying as Black and low rates of acceptance of COVID-19 vaccine trial participation and actual vaccination. The findings suggest that partnerships between health care and other sectors to build trust and promote vaccination may benefit from socially and culturally responsive strategies that acknowledge and address racial/ethnic health care disparities and historical and contemporary experiences of racism.

## Key Points

**Question:**

Is there an association between race/ethnicity and rejection of COVID-19 vaccine trial participation and vaccine uptake in the US, and does racial/ethnic group–based medical mistrust mediate this association?

**Findings:**

In this survey study of 1835 adults in Michigan, Black participants reported the greatest medical mistrust among the racial/ethnic groups surveyed. Analysis of path models revealed significantly greater COVID-19 vaccine trial and uptake rejection among Black participants, which was partially mediated by medical mistrust.

**Meaning:**

The findings suggest that racial/ethnic group–based medical mistrust may partially explain the association between Black race/ethnicity and rejection of COVID-19 vaccine trial participation and uptake, potentially informing socially and culturally responsive efforts to promote COVID-19 vaccination in this group.

## Teaser

This survey study of adults residing in Michigan during June to December 2020 examines associations between race/ethnicity, medical mistrust within racial/ethnic groups, and willingness to participate in COVID-19 vaccine trials or to receive a COVID-19 vaccine.

## Introduction

In the US, there have been more than 29.8 million confirmed COVID-19 cases and more than 542 400 deaths from this disease.^1^ Nationwide, racial and ethnic disparities have been substantial. Data show that Black individuals represent 22% of cases to date while only representing 13% of the US population.^2^ Trends have been similar in Michigan, where COVID-19 incidence has been higher among Black individuals (48 443 per 1 000 000 population) compared with White individuals (45 427 per 1 000 000 population) and all other racial/ethnic groups.^3^ COVID-19–related mortality has also been higher among Black individuals (2328 per 1 000 000 population) compared with White individuals (1329 per 1 000 000 population) and other racial/ethnic groups. Of note, Black individuals account for 14% of the population in Michigan but have represented 23% of COVID-19–related deaths to date.^4^

Vaccination against COVID-19 has been widely viewed as an essential component of a strategic plan to control community spread of COVID-19. However, public acceptance of a COVID-19 vaccine has varied. For example, in 1 nationally representative sample of 991 adults,^5^ participants were asked, “When a vaccine for the coronavirus becomes available, will you get vaccinated?” In this sample, 10.8% reported “no” and 31.6% reported “not sure”; Black participants were significantly more likely to report “no” or “not sure” compared with White participants. Among those who reported no intention to be vaccinated, 32.5% cited lack of trust as a reason, including distrust of vaccines, government, pharmaceutical companies, and vaccine development or testing processes. The study did not explicitly examine lack of trust across racial and ethnic groups. However, there are compelling reasons why Black individuals in the US in particular would distrust a COVID-19 vaccine, and these reasons are rooted in racism. These include implicit bias within health care systems and among health care professionals that may be associated with lower quality of care and worse health outcomes among Black individuals in the US^6^ and the rapid development and promotion of a COVID-19 vaccine within a sociopolitical climate that many Black individuals in the US perceive as hostile to them.^7,8^

This survey study focused on examining the acceptability of a hypothetical COVID-19 vaccine. The study also assessed acceptability of a hypothetical COVID-19 vaccine research trial. Lackland et al^9^ noted that the participation of underrepresented racial/ethnic groups in COVID-19 trials is essential for external validity and translatability of results. Furthermore, the enrollment of members of these groups in trials may potentially increase acceptability of the vaccine among similar other individuals by helping to establish vaccination as an emerging group norm. In the current study, we hypothesized that (1) self-reported Black race would be associated with greater rejection of both COVID-19 vaccine trials and vaccine uptake and (2) racial/ethnic group–based medical mistrust would mediate the association between Black race and greater vaccine rejection. Illuminating these associations may assist in the understanding of the layered vaccine-related apprehensions of a population particularly burdened by COVID-19 and in the promotion of equitable access to an effective vaccine.

## Methods

### Participants and Procedures

In this survey study, participants included 1835 adult Michigan residents aged 18 years or older who responded to at least 1 of 2 survey items assessing agreement to participate in a COVID-19 vaccine trial and to receive a COVID-19 vaccine. A purposive sampling strategy was used that relied on survey dissemination through 9 community-based organizations (CBOs) throughout Michigan that were already formal community research partners with the study’s lead institution. These CBOs included social service agencies targeting parents, older individuals, and LGBTQ (lesbian, gay, bisexual, transgender, queer) populations as well as rural communities, faith-based organizations, and federally qualified health centers. A link to an online survey was disseminated through these CBOs and through communications and marketing networks of hospitals affiliated with the lead academic institution. Participants also had the option of completing the survey via telephone interview. Participants were enrolled from June to December 2020 and received $10 for participating. This study was reviewed and approved by Wayne State University’s institutional review board (IRB), which granted a waiver of oral and signed informed consent because survey dissemination online could not practicably be carried out without this alteration. All participants were provided with an IRB-approved study information sheet before survey administration, representing an unsigned form of passive consent in which the participant indicated consent by proceeding with this minimal-risk task. The study followed the American Association for Public Opinion Research (AAPOR) reporting guideline for survey studies and met the expectations for the reporting of recruitment and participation outcomes, institutional research standards and ethics, and best practices.

### Assessment

Participants’ level of agreement with participation in a vaccine trial was assessed with 1 item adapted from the work of Jacobsen et al^10^: “If you were asked today to participate in a research study to test a COVID-19 vaccine, would you agree to participate?” A similar item was asked regarding vaccine uptake: “If you were offered a coronavirus vaccine that had been approved by the US FDA [US Food and Drug Administration] today, would you agree to be vaccinated?” For both items, responses were based on a Likert-type scale with available responses of “definitely yes” (1), “probably yes” (2), “neither yes or no” (3), “probably no” (4), or “definitely no” (5). Group-based medical mistrust was measured using the 6-item suspicion subscale of the Group-Based Medical Mistrust Scale,^11^ a 12-item scale assessing suspicion of mainstream health care systems and professionals and of the treatment provided to individuals of the respondent’s racial/ethnic group. Responses were based on a Likert-type scale ranging from 1 (strongly disagree) to 5 (strongly agree), with higher scores representing greater mistrust. Reliability in the current sample was high (Cronbach α = .94).

Sociodemographic variables were also assessed, including age, gender, income level, essential worker status, and race/ethnicity. Participants were asked to self-identify based on categories provided by study investigators: White; Black or African American; Arab, Chaldean, Middle Eastern, or North African (MENA); Hispanic; Asian; and multiracial or other groups.

### Statistical Analysis

Sociodemographic characteristics, including medical mistrust, were summarized by count and percentage for categorical variables and mean (SD) for continuous variables. The association between race/ethnicity and medical mistrust was assessed by 1-way analysis of variance.

The study hypotheses addressed the associations between race/ethnicity, medical mistrust, and outcomes as outlined in our path model (Figure). As an analytic approach, path analysis offered an efficient strategy to test the fit of this proposed model by examining a covariance structure consistent with hypothesized associations among model constructs. We used a separate path analysis for each outcome, fit with the lavaan package^12,13^ using R, version 3.6.3 (R Project for Statistical Computing).^14^ We specified paths from the racial/ethnic group and covariates to outcomes (ie, substantive associations and covariate controls) and paths from the racial/ethnic group to medical mistrust (ie, a direct association between race/ethnicity and the mediator). Consequently, the model included estimates of direct associations between racial/ethnic groups and mistrust, separately, on the outcomes, as well as indirect associations between racial/ethnic groups and outcomes that were mediated by mistrust. Products of relevant coefficients (Figure) and associated bootstrap SEs indicated whether the indirect associations were statistically significant.

**Figure.**
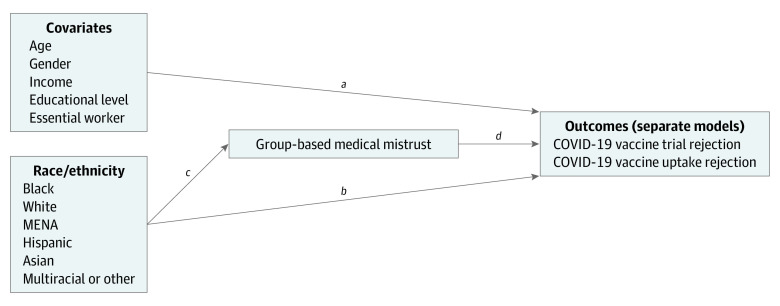
Path Model The product of paths from the racial/ethnic group to mistrust and mistrust to outcome (*c* × *d*) indicates indirect associations. MENA indicates Arab, Chaldean, Middle Eastern, or North African.

Given this study’s cross-sectional convenience sample, the indirect associations cannot be interpreted as causal paths; rather, they should be interpreted as evidence that a model construct (eg, racial/ethnic group) may simultaneously share a significant association with a separate model construct (eg, mistrust) and the substantive model outcome (eg, rejection).^15^

The outcomes were coded such that higher scores indicated greater rejection of vaccine trial participation and vaccine uptake. Age was entered as a continuous variable. Racial/ethnic categories were effect coded so that the model’s coefficients represented a group’s deviation from the adjusted overall mean estimate for the outcomes. The multiracial or other group was used as the effect-coded reference group (ie, the coefficient did not appear in the model) to examine deviations for the racial/ethnic groups that were more commonly identified and investigated. However, effect-coded coefficients indicated deviation from the overall mean estimate and not from the reference group. All other categorical variables (gender, educational level, income level, and essential worker status) were dummy coded such that the coefficients represented deviation from a reference group’s mean estimate. Statistical significance was set at a 2-tailed *P* < .05.

## Results

Table 1 presents sociodemographic characteristics. Among the 1835 individuals who participated in the survey, the mean (SD) age was 49.4 (17.9 years; range, 18-94 years), with participants aged 40 to 64 years representing approximately 42% of the sample. A total of 1455 respondents (79%) were female, 361 (20%) were male, and 19 (1%) identified as other gender. The study sample was somewhat evenly divided across the 3 specified annual income levels (28%-30%), with 211 participants (11%) choosing to withhold information about income. Of the total respondents, 962 (52%) were White individuals and 394 (21%) were Black individuals. The next largest racial/ethnic group was individuals who identified as multiracial or other (205 [11%]), followed by MENA (93 [5%]), Hispanic (84 [5%]), and Asian (97 [5]) respondents. Of the overall sample, 348 (19%) identified as essential workers.

**Table 1.** Sociodemographic Characteristics Identified by the Study Participants

Characteristic	Participants, No. (%) (N = 1835)^a^
Age, y	
18-39	603 (33)
40-64	772 (42)
≥65	460 (25)
Gender	
Male	361 (20)
Female	1455 (79)
Other	19 (1)
Race/ethnicity	
White	962 (52)
Black	394 (21)
MENA	93 (5)
Hispanic	84 (5)
Asian	97 (5)
Other^b^	205 (11)
Annual income, $	
0-34 999	517 (28)
35 000-74 999	553 (30)
≥75 000	554 (30)
Prefer not to answer	211 (11)
Essential worker	
No	1487 (81)
Yes	348 (19)
Educational level	
High school or less	276 (15)
Some college or associate’s degree	617 (34)
Bachelor’s degree	489 (27)
Graduate degree	445 (24)
Prefer not to answer	10 (0.5)

Abbreviation: MENA, Arab, Chaldean, Middle Eastern, or North African.

^a^ Percentages may not sum to 100 owing to rounding.

^b^ Other included American Indian or Alaskan Native, Native Hawaiian or other Pacific Islander, multiracial, or race/ethnicity not specified.

The mean (SD) medical mistrust score among all participants was 1.84 (0.91; range, 1.0-5.0). Black participants had the highest mistrust scores (mean [SD] score, 2.35 [0.96]) compared with other groups compared with other racial/ethnic groups (mean [SD] for the total sample, 1.83 [0.91]), followed by those who identified as Hispanic (mean [SD] score, 2.22 [0.95]). All scores across groups were significantly different (Table 2).

**Table 2.** Medical Mistrust by Race/Ethnicity

Racial/ethnic group	Medical mistrust score, mean (SD)^a^
All	1.83 (0.91)
White	1.52 (0.71)
Black	2.35 (0.96)
MENA	1.77 (0.90)
Hispanic	2.22 (0.95)
Asian	2.10 (1.01)
Other^b^	2.11 (1.01)

Abbreviation: MENA, Arab, Chaldean, Middle Eastern, or North African.

^a^ *P* < .001 for all based on 1-way analysis of variance.

^b^ Other included American Indian or Alaskan Native, Native Hawaiian or other Pacific Islander, multiracial, or race/ethnicity not specified.

In response to the item asking about trial participation (n = 1827), response rates were as follows: “definitely yes,” 8%; “probably yes,” 17%; “neither yes or no” (unsure), 11%; “probably no,” 27%; and “definitely no,” 37%. Table 3 shows the proportion of the total sample and of each racial/ethnic group that responded “definitely or probably no or unsure” vs “definitely or probably yes” to whether they would participate in a COVID-19 vaccine trial. A total of 1376 participants (75%) had responses consistent with greater rejection; differences were observed across race/ethnicity. In response to the item regarding vaccination (n = 1815), response rates were as follows: “definitely yes,” 20%; “probably yes,” 28%; “neither yes or no,” 12%; “probably no,” 19%; and “definitely no,” 21%. Table 3 also shows the proportion of the total sample and of each racial/ethnic group that responded “definitely or probably no or unsure” vs “definitely or probably yes” to the question of whether they would receive a COVID-19 vaccine. Participants were divided approximately evenly between the 2 categories.

**Table 3.** COVID-19 Vaccine Trial and Uptake Willingness by Race/Ethnicity

Response	Participants, No. (%)						
	All	White	Black	MENA	Hispanic	Asian	Multiracial or other
Would participate in a vaccine trial							
Definitely no, probably no, or unsure	1376 (75)	669 (70)	345(88)	76 (82)	57 (68)	67 (70)	162 (80)
Definitely yes or probably yes	451 (25)	290 (30)	45 (12)	17 (18)	27 (32)	29 (30)	43 (21)
Would receive a vaccine							
Definitely no, probably no, or unsure	945 (52)	410 (43)	279 (72)	57 (62)	49 (58)	34 (36)	116 (57)
Definitely yes or probably yes	870 (48)	545 (57)	108 (28)	35 (38)	35 (42)	61 (64)	86 (43)

Abbreviation: MENA, Arab, Chaldean, Middle Eastern, or North African.

Table 4 shows results of path analysis for vaccine trial rejection. Model indices indicated a decent model fit (*N* listwise, 1571; χ^2^_8_, 47.51; *P* < .001; root mean square error of approximation [RMSEA], 0.06 [90% CI, 0.04-0.07]; comparative fit index [CFI], 0.91; standardized root mean square residual [SRMR], 0.02); measures indicating suboptimal fit (ie, χ^2^ and CFI) were likely attributable to the large sample size. Among covariates, results indicated significantly greater trial rejection among female individuals (*B* [SE], 0.62 [0.08]; *P* < .001) and those with higher mistrust scores (*B* [SE], 0.13 [0.04]; *P* = .001) and lower rejection among those with a graduate (*B* [SE], −0.31 [0.12]; *P* = .008) or college (*B* [SE], −0.23 [0.11]; *P* = .04) educational level compared with a high school educational level. Compared with overall mean rejection, there was significantly more rejection of participation in a vaccine trial among Black participants (*B* [SE], 0.51 [0.08]; *P* < .001) and lower rejection among Hispanic (*B* [SE], −0.29 [0.13]; *P* = .02), Asian (*B* [SE], −0.27 [0.13]; *P* = .04), and White (*B* [SE], −0.13 [0.07], *P* = .05) participants. The results further indicated that among Black (*B* [SE], 0.04 [0.01]; *P* = .003) and White (*B* [SE], −0.06 [0.02]; *P* = .001) participants, the association between race/ethnicity and trial rejection was partially mediated by medical mistrust. In addition, although no direct association was found between race/ethnicity and trial rejection among MENA participants, there was a significant indirect association (*B* [SE], −0.03 [0.02]; *P* = .04) owing to the significant association between identifying as MENA and mistrust *(B* [SE], −0.24 [0.09]; *P* = .005), suggesting that mistrust mediated the association between trial rejection and identifying as MENA.

**Table 4.** Path Analysis Modeling Vaccine Trial and Vaccine Uptake Rejection

Variable	Vaccine trial rejection		Vaccine uptake rejection	
	*B* (SE)	*P* value	*B* (SE)	*P* value
**Direct association with outcome**				
Age	0.00 (0.00)	NA	0.00 (0.00)	NA
Gender				
Male	1 [Reference]	NA	1 [Reference]	NA
Female	0.62 (0.08)	<.001	0.57 (0.08)	<.001
Race/ethnicity^a^				
Black	0.51 (0.08)	<.001	0.51 (0.08)	<.001
MENA	0.16 (0.13)	.22	0.19 (0.14)	.18
Hispanic	−0.29 (0.13)	.02	−0.02 (0.13)	.86
Asian	−0.27 (0.13)	.04	−0.63 (0.14)	<.001
White	−0.13 (0.07)	.05	−0.20 (0.07)	.005
Annual income, $				
0-35 000	1 [Reference]	NA	1 [Reference]	NA
35 000-75 000	−0.03 (0.08)	.75	−0.21 (0.09)	.02
≥75 000	0.05 (0.09)	.55	−0.17 (0.09)	.07
Essential worker				
No	1 [Reference]	NA	1 [Reference]	NA
Yes	0.03 (0.08)	.75	0.26 (0.09)	.003
Educational level				
High school diploma or less	1 [Reference]	NA	1 [Reference]	NA
Some college	−0.19 (0.10)	.07	−0.11 (0.11)	.30
College degree	−0.23 (0.11)	.04	−0.15 (0.12)	.20
Graduate degree	−0.31 (0.12)	.008	−0.25 (0.12)	.04
Medical mistrust	0.13 (0.04)	.001	0.21 (0.04)	<.001
**Direct association between race/ethnicity and outcome**				
Black	0.32 (0.05)	<.001	0.33 (0.05)	<.001
MENA	−0.24 (0.09)	.005	−0.24 (0.09)	.005
Hispanic	0.21 (0.08)	.01	0.21 (0.08)	.01
Asian	0.06 (0.08)	.49	0.05 (0.09)	.53
White	−0.49 (0.04)	<.001	−0.49 (0.04)	<.001
*R*^2^				
Outcome^b^	0.11^c^	NA	0.14^c^	NA
Medical mistrust^d^	0.15^c^	NA	0.15^c^	NA
**Indirect association between race/ethnicity and outcome**^e^				
Black	0.04 (0.01)	.003	0.07 (0.02)	<.001
MENA	−0.03 (0.02)	.04	−0.05 (0.02)	.01
Hispanic	0.03 (0.01)	.06	0.04 (0.02)	.02
Asian	0.01 (0.01)	.56	0.01 (0.02)	.54
White	−0.06 (0.02)	.001	−0.10 (0.02)	<.001

Abbreviations: MENA, Arab, Chaldean, Middle Eastern, or North African; NA, not applicable.

^a^ Effect-coded coefficients indicate deviation from the overall mean, not from a reference group.

^b^ Variance accounted for by covariates, race, and mistrust (paths *a*, *b*, and *d* in the path model [Figure]).

^c^ Estimates are *R*^2^.

^d^ Variance accounted for by race/ethnicity (path *c* in the path model [Figure]).

^e^ Indirect associations were calculated as the product of paths from racial/ethnic group to mistrust and mistrust to outcome (Figure).

Table 4 also shows results of path analysis for vaccine uptake rejection. Model indices indicated decent model fit (*N* listwise, 1564; χ^2^_8_, 48.85; *P* < .001; RMSEA, 0.06 [90% CI, 0.04-0.07]; CFI, 0.92; SRMR, 0.02). Among covariates, there was greater vaccine uptake rejection among female individuals (*B* [SE], 0.57 [0.08]; *P* < .001), essential workers *(B* [SE], 0.26 [0.09]; *P* = .003), and those with higher mistrust scores *(B* [SE], 0.21 [0.04]; *P* < .001). There was less rejection among those in the $35 000 to $75 000 income category (*B* [SE], −0.21 [0.09]; *P* = .02) compared with those in the $0 to $35 000 income category and among those with graduate degrees (*B* [SE], −0.25 [0.12]; *P* = .04) compared with those with a high school educational level. Compared with the overall mean rejection, there was greater rejection among Black participants (*B* [SE], 0.51 [0.08]; *P* < .001) and less rejection among Asian (*B* [SE], −0.63 [0.14]; *P* < .001) and White (*B* [SE], −0.20 [0.07]; *P* = .005) participants. The race/ethnicity associations were partially mediated by mistrust among Black (*B* [SE], 0.07 [0.02]; *P* < .001) and White (*B* [SE], −0.10 [0.02]; *P* < .001) participants. Indirect associations were present among MENA (*B* [SE], −0.05 [0.02]; *P* = .01) and Hispanic (*B* [SE], 0.04 [0.02]; *P* = .02) participants, suggesting that any association between these groups and rejection was mediated by mistrust.

## Discussion

Broad vaccine acceptance is generally regarded as critical to the long-term containment of COVID-19 in the US. The findings of the current study may be useful and formative for researchers, clinicians, and public health leaders to better understand the associations between racial/ethnic group-based medical mistrust and the willingness of diverse racial/ethnic groups to participate in COVID-19 vaccine trials and to accept a federally approved COVID-19 vaccine.

Of note, most participants in this study indicated low willingness or refusal to participate in a COVID-19 clinical trial to test the efficacy of a vaccine, with rejection highest among Black participants, followed by those who identified as MENA. Vaccine uptake rejection was lower, with half of participants indicating responses consistent with rejection and Black participants reporting the most refusal, followed by individuals who identified as MENA and Hispanic. These findings are consistent with other recent studies documenting widespread COVID-19 vaccine rejection in the US.^5,16^ Although those studies showed differences across race and ethnicity, the current study is, to our knowledge, the first to investigate the role of racial/ethnic group–based medical mistrust in acceptance and rejection of COVID-19 vaccines. Specifically, medical mistrust partially mediated the association between Black race/ethnicity and refusal to participate in vaccine trials or receive a vaccine, suggesting that general suspicion of mainstream health care professionals and systems may be associated with this group’s rejection of the vaccine.

A recent commentary by Warren and colleagues^17^ offered insight relevant to these findings, citing the “deep and justified lack of trust” that many Black individuals in the US have of health care systems and clinical research. These authors state, “This distrust is often traced to the legacy of the infamous syphilis study at Tuskegee, in which investigators withheld treatment from hundreds of Black men in order to study the natural history of the disease. But the distrust is far more deeply rooted, in centuries of well-documented examples of racist exploitation by American physicians and researchers.” Of importance, mistrust may be rooted in contemporary health care experiences. Such experiences were revealed in the results of a 2020 Kaiser Family Foundation survey of 1700 adults that included nearly 800 Black individuals in the US.^18^ The findings showed that 45% of Black respondents reported at least 1 of 6 negative experiences with a health care professional (eg, the health care professional assumed something about them without asking, talked down to them or did not treat them with respect, or did not believe they were telling the truth), and 36% reported believing that they would have received better medical care if they had belonged to a different race/ethnicity. Such findings support the notion that mistrust is associated with perceptions of past injustices as well as present-day experiences.

The current study also offers some insight into vaccine rejection among other racial/ethnic groups. The results showed that medical mistrust within racial/ethnic groups was associated with vaccine rejection in the overall sample regardless of race/ethnicity. Furthermore, although direct associations were not found between identifying as MENA or Hispanic and vaccine uptake rejection, significant indirect associations through mistrust were identified, suggesting that medical mistrust may inform low acceptance within these groups. These findings warrant further investigation, especially in MENA communities at risk of exclusion in COVID-19 vaccine promotion efforts. MENA populations are typically underrepresented or unrepresented in behavioral research on health disparities because they are not formally recognized by the US government as a minority group and as distinct from White individuals. However, there is increasing evidence that this group, particularly Arab individuals in the US, experiences numerous barriers to care, including group-based medical mistrust and perceived discrimination.^19,20^

As the US continues nationwide COVID-19 vaccine dissemination and campaigns to promote vaccination, Warren and colleagues^17^ suggest that large-scale coordinated grassroots efforts will be required to reduce vaccine rejection and increase acceptance. These efforts should include meaningful partnerships between health care and academic institutions with trusted community stakeholders and CBOs representing communities of color along with extensive investment in building trust across the spectrum from vaccine development to dissemination. This approach is endorsed by Ojikutu et al,^21^ who recommend a range of strategies for building trust through authentic community investment, including financial investments in CBOs that serve as vaccine research partners; funding the involvement of CBOs in vaccine uptake interventions; providing in-kind resources to build capacity within CBOs; increasing transparency of government contracts for vaccine manufacturing and distribution and mandating contracts with Black-owned businesses; and supporting career development among scientists and health care professionals of color. We further recommend that all efforts acknowledge and address, in their development and/or implementation, the historical and contemporary experiences of racism in the health care domain and beyond among Black individuals in the US. Unless efforts to promote COVID-19 vaccination are contextualized within these experiences, they may not fully address salient concerns of this group.

### Limitations

This study has limitations. A probability sampling strategy was not used, and data were not collected from a random sample of the Michigan population. However, we attempted to compensate for this through the use of a maximum variation sampling strategy to approximate the potential responses of a population-based sample. Another limitation is that despite this approach, those who identified as male only represented 20% of the sample, reducing the generalizability of the findings. In addition, the study only assessed mistrust and did not include other variables found to be associated with vaccine rejection in the extant literature, such as concerns about vaccine safety and efficacy, which may be particularly relevant to COVID-19 vaccines in light of the unprecedented speed at which COVID-19 vaccines were developed and advanced to human trials.^22^ Future work should not only assess mistrust but also assess other variables known to be associated with vaccine acceptance.

## Conclusions

In this survey study, racial/ethnic group-based medical mistrust partially mediated the association between Black race and lower acceptance of COVID-19 vaccine trial participation and actual vaccination. The findings suggest that partnerships between health care and other sectors to build trust and promote vaccination may benefit from socially and culturally responsive strategies that acknowledge and address racial/ethnic disparities in health care and historical and contemporary experiences of racism among Black individuals in the US.

## References

[1] Center for Systems Science and Engineering at Johns Hopkins University. Coronavirus COVID-19 global cases by the Center for Systems Science and Engineering (CSSE) at Johns Hopkins University (JHU). Accessed March 23, 2021. https://coronavirus.jhu.edu/us-map

[2] Stokes EK, Zambrano LD, Anderson KN, . Coronavirus disease 2019 case surveillance—United States, January 22–May 30, 2020. MMWR Morb Mortal Wkly Rep. 2020;69(24):759-765. doi:10.15585/mmwr.mm6924e2 32555134 PMC7302472

[3] State of Michigan. Coronavirus: Michigan data. Accessed March 22, 2021. https://www.michigan.gov/coronavirus/0,9753,7-406-98163_98173---,00.html

[4] COVID Tracking Project. The COVID racial data tracker. Accessed March 23, 2021. https://covidtracking.com/race

[5] Fisher KA, Bloomstone SJ, Walder J, Crawford S, Fouayzi H, Mazor KM. Attitudes toward a potential SARS-CoV-2 vaccine: a survey of U.S. adults. Ann Intern Med. 2020;173(12):964-973. doi:10.7326/M20-3569 32886525 PMC7505019

[6] Maina IW, Belton TD, Ginzberg S, Singh A, Johnson TJ. A decade of studying implicit racial/ethnic bias in healthcare providers using the implicit association test. Soc Sci Med. 2018;199:219-229. doi:10.1016/j.socscimed.2017.05.009 28532892

[7] Abutaleb Y, McGinley L, Johnson CY. How the “deep state” scientists vilified by Trump helped him deliver an unprecedented achievement. *Washington Post*. Published December 14, 2020. Accessed January 5, 2021. https://www.washingtonpost.com/health/2020/12/14/trump-operation-warp-speed-vaccine/

[8] Kendi IX. The end of denial. The Atlantic. Published August 5, 2020. Accessed January 5, 2021. https://www.theatlantic.com/press-releases/archive/2020/08/ibram-x-kendi-on-the-end-of-denial/614962/

[9] Lackland DT, Sims-Robinson C, Jones Buie JN, Voeks JH. Impact of COVID-19 on clinical research and inclusion of diverse populations. Ethn Dis. 2020;30(3):429-432. doi:10.18865/ed.30.3.429 32742146 PMC7360182

[10] Jacobsen PB, Wells KJ, Meade CD, . Effects of a brief multimedia psychoeducational intervention on the attitudes and interest of patients with cancer regarding clinical trial participation: a multicenter randomized controlled trial. J Clin Oncol. 2012;30(20):2516-2521. doi:10.1200/JCO.2011.39.5186 22614993 PMC4577714

[11] Thompson HS, Valdimarsdottir HB, Winkel G, Jandorf L, Redd W. The Group-Based Medical Mistrust Scale: psychometric properties and association with breast cancer screening. Prev Med. 2004;38(2):209-218. doi:10.1016/j.ypmed.2003.09.041 14715214

[12] Rosseel Y. lavaan: an R package for structural equation modeling. J Stat Softw. 2012;48(2):1-36. doi:10.18637/jss.v048.i02

[13] Rosseel Y. The Lavaan Tutorial. Ghent University; 2015.

[14] *R: A Language and Environment for Statistical Computing.* R Foundation for Statistical Computing; 2016.

[15] Streiner DL. Finding our way: an introduction to path analysis. Can J Psychiatry. 2005;50(2):115-122. doi:10.1177/070674370505000207 15807228

[16] Kreps S, Prasad S, Brownstein JS, . Factors associated with US adults’ likelihood of accepting COVID-19 vaccination. JAMA Netw Open. 2020;3(10):e2025594-e2025594. doi:10.1001/jamanetworkopen.2020.25594 33079199 PMC7576409

[17] Warren RC, Forrow L, Hodge DA Sr, Truog RD. Trustworthiness before trust—COVID-19 vaccine trials and the Black community. N Engl J Med. 2020;383(22):e121. doi:10.1056/NEJMp2030033 33064382

[18] Hamel L, Lopes L, Muñana C, Artiga S, Brodie M. Race, Health, and COVID-19: The Views and Experiences of Black Americans. Key Findings from the KFF/Undefeated Survey on Race and Health. Kaiser Family Foundation; 2020.

[19] Jaffee K, Cohen M, Azaiza F, Hammad A, Hamade H, Thompson H. Cultural barriers to breast cancer screening and medical mistrust among Arab American women. J Immigr Minor Health. 2021;23(1):95-102. doi:10.1007/s10903-020-01019-0 32451692

[20] Abuelezam NN, El-Sayed AM, Galea S. The health of Arab Americans in the United States: an updated comprehensive literature review. Front Public Health. 2018;6:262. doi:10.3389/fpubh.2018.00262 30255009 PMC6141804

[21] Ojikutu BO, Stephenson KE, Mayer KH, Emmons KM. Building trust in COVID-19 vaccines and beyond through authentic community investment. Am J Public Health. 2021;111(3):366-368. doi:10.2105/AJPH.2020.306087 33301352 PMC7893367

[22] Domek GJ, O’Leary ST, Bull S, . Measuring vaccine hesitancy: field testing the WHO SAGE Working Group on Vaccine Hesitancy survey tool in Guatemala. Vaccine. 2018;36(35):5273-5281. doi:10.1016/j.vaccine.2018.07.046 30061026 PMC6145454

